# Epigallocatechin-3-gallate (EGCG) inhibits the migratory behavior of tumor bronchial epithelial cells

**DOI:** 10.1186/1465-9921-9-33

**Published:** 2008-04-21

**Authors:** Salma Hazgui, Arnaud Bonnomet, Béatrice Nawrocki-Raby, Magali Milliot, Christine Terryn, Jérôme Cutrona, Myriam Polette, Philippe Birembaut, Jean-Marie Zahm

**Affiliations:** 1 INSERM, UMRS903, Reims, F-51092 France; 2 Univ Reims Champagne Ardenne, IFR53, Reims, F-51100 France; 3 CHU Reims, Hôpital Maison Blanche, Laboratoire Pol Bouin, Reims, F-51092 France

## Abstract

**Background:**

Many studies associated the main polyphenolic constituent of green tea, (-)-Epigallocatechin-3-gallate (EGCG), with inhibition of cancers, invasion and metastasis. To date, most of the studies have focused on the effect of EGCG on cell proliferation or death. Since cell migration is an important mechanism involved in tumor invasion, the aim of the present work was to target another approach of the therapeutic effect of EGCG, by investigating its effect on the cell migratory behavior.

**Methods:**

The effect of EGCG (at concentrations lower than 10 μg/ml) on the migration speed of invasive cells was assessed by using 2D and 3D models of cell culture. We also studied the effects of EGCG on proteinases expression by RT-PCR analysis. By immunocytochemistry, we analyzed alterations of vimentin organization in presence of different concentrations of EGCG.

**Results:**

We observed that EGCG had an inhibitory effect of cell migration in 2D and 3D cell culture models. EGCG also inhibited MMP-2 mRNA and protein expression and altered the intermediate filaments of vimentin.

**Conclusion:**

Taken together, our results demonstrate that EGCG is able to inhibit the migration of bronchial tumor cells and could therefore be an attractive candidate to treat tumor invasion and cell migration.

## Background

Cell migration is a prerequisite for cancer invasion and metastasis. Much of the focus on the therapeutic treatment of cancer has involved compounds that target cell proliferation and subsequent cell death. However, targeting migration is another approach that has not been extensively pursued but holds promise for alternative means of therapy [1].

Tea (*Camellia sinensis*) is a popular beverage worldwide. (-)-Epigallocatechin-3-gallate (EGCG), the main polyphenolic constituent of green tea, has been shown to have association with prevention of cancer development, metastasis, invasion and angiogenesis [2]. To date, most of the studies have focused on the effect of EGCG on cell proliferation or death. EGCG has been shown to induce apoptosis in many human cell lines: human lymphoid leukemia cells [3], prostate cancer cell lines [4], human epidermoid carcinoma A431 cells [5], breast carcinoma MCF-7 cells [6], melanoma cells [7] and pancreatic cancer cells [8]. Previous studies demonstrated that it has a selective apoptotic effect in tumor cells compared with normal cells [9]. This polyphenolic component has also an inhibitory effect on angiogenesis that is an important process in tumor growth [10].

The acquisition of an invasive phenotype by epithelial cells implicates a series of changes altering their differentiation [11]. Components of the extracellular matrix play a fundamental role in the process of tumor invasion. Extensive studies in the last decade have revealed that matrix metalloproteases (MMP) are frequently overexpressed in most forms of human tumor [12,13] and are implicated in the destruction of the extracellular matrix, thus facilitating tumor invasion [14,14,15]. EGCG has inhibitory effects on MMP-2 and MT1-MMP in glioblastoma cells [16], reduces MT1-MMP activity in an invasive human fibrosarcoma cell line [17] and induces repression of MMP-9 expression in lung carcinoma cell invasion [18]. It reduces cancer cell proliferation and migration by a combination with ascorbic acid [19], by reducing VEGF production [20]. EGCG also downregulates ephrin-A1-mediated endothelial cell migration [21] and melanoma and pancreatic cancer growth and metastasis [22,23]. Using a wound healing assay, Siddiqui et al [24] demonstrated that co-treatment of prostate carcinoma cells with EGCG and TNF-related apoptosis-inducing ligand led to a decrease in cell migration. However, the studies dealing with cell migration were mostly performed by using *in vitro *models by which cell migration was evaluated by using the Boyden chamber technique, or referred to qualitative rather than quantitative data. Our aim was to use *in vitro *models of cell migration and to study the EGCG effects on cell movement by analyzing the dynamic cell behavior of a tumor epithelial bronchial cell line. We used a two-dimensional (2D) model of cell dispersion [25] and a three-dimensional (3D) model of cell migration to mimic conditions similar to those observed in vivo during tumor invasion [26]. In parallel we analyzed the effect of EGCG on protease expression and vimentin organization.

## Methods

### Cell lines

The BZR human bronchial cell line used in our study [27] was derived from normal human bronchial cells immortalized after transfection with the SV40 large T-antigen gene and infected with the v-Ha-ras oncogene. This cell line displays an invasive potential *in vitro *and tumorigenicity and metastatic ability in athymic nude mice. Cells were cultured in a 5% CO_2 _fully humidified atmosphere at 37°C in Dulbecco modified Eagle's medium (DMEM) (Gibco BRL, Grand Island, USA) supplemented with penicillin, streptomycin (Eurobio, les Ulis, France) and 10% fetal calf serum (Gibco BRL). Human epithelial MCF10A cells were obtained from the American Type culture collection and cultured in HAM F12 and DMEM (1:3 v/v) supplemented with 20 μg/ml of adenine, 5 μg/ml of insulin, 0.5 μg/ml of hydrocortisone, 2 ng/ml of EGF, 5 μg/ml of transferrin, 1.5 ng/ml of triiodothyronine and 10% fetal calf serum. EGCG was purchased from Sigma Aldrich (Saint-Quentin Fallavier, France) and stored at 4°C.

### Effect of EGCG on cell death

The BZR cell line was plated at 1 × 10^5 ^cells/ml and after 2 days of culture, the medium was removed from the culture plates and replaced with serum free medium with 5, 10, or 20 μg/ml of EGCG. After 18 h of cell interaction with EGCG, the fluorescent probe propidium iodide (Invitrogen, Cergy Pontoise, France), diluted at 20 mM in the culture medium, was used to visualize the cell death. Fluorescent images were recorded using an inverted microscope (Zeiss Axiovert 200, Le Pecq, France). From the fluorescent images, the mean grey level, proportional to the number of dead cells, was measured and reported as cell death index.

### 2D cell Migration Assay

The BZR cell line was plated at 10^3 ^cells/ml and after 2 days of culture, the medium was removed from the culture plates and replaced with serum free medium with 5 μg/ml or 7.5 μg/ml of EGCG. Cell migration experiments were performed using an inverted microscope (Axiovert 200, Zeiss, Le Pecq, France) equipped with a small transparent environmental chamber (Climabox, Zeiss) with 5% CO2 in air at 37°C. The microscope was driven by the Metamorph software (Roper Scientific, Evry, France) and images of the cells were recorded every 15 min for 18 hours with a CCD camera (Coolsnap, Roper Scientific) at 20× magnification The migration speed of BZR cell line was determined as previously described by Zahm et al [28].

### 3D cell migration assay

**Figure 1:**
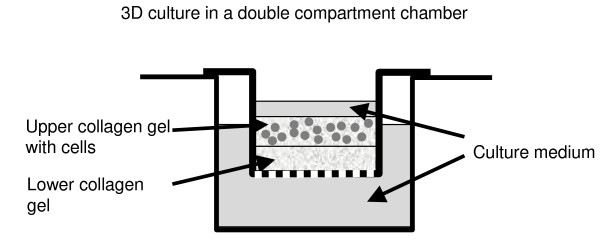
**Representation of the 3D culture model**. A collagen gel layer was prepared by mixing 400 μl type I collagen at 2 mg/ml with 150 μl RPMI culture medium 5 fold concentrated, 15 μl NaOH 1 N and 100 μl DMEM with 10% fetal calf serum. To form the first layer of the microenvironment, 150 μl of this mixture was deposited on the membrane of a double compartment chamber (Transwell) and polymerized for 30 minutes at 37°C. A second collagen gel layer was formed by mixing 400 μl type I collagen at 2 mg/ml with 150 μl RPMI 5×, 15 μl NaOH 1N, 100 μl DMEM with 10% fetal calf serum and BZR cell suspension at 13 × 10^4 ^cells/ml. 150 μl of this mixture was added over the first layer and 1.5 ml of DMEM was placed into the basal compartment of the chamber which was thereafter maintained for 24 hours at 37°C.

Type I collagen gel was extracted from rat tails according to the method described by Chambard et al [29]. To visualize cells in a 3D model, we have developed a microenvironment model that consists of a two-layer type I collagen gel (figure 1). The first collagen gel layer was prepared by mixing 400 μl type I collagen at 2 mg/ml with 150 μl RPMI 5×, 15 μl NaOH 1 N and 100 μl DMEM with 10% fetal calf serum. To form the first layer of the microenvironment, 150 μl of this mixture was deposited on the membrane of a double compartment chamber (Transwell, Corning, Acton, MA) and polymerized for 30 minutes at 37°C. A second collagen gel layer was formed by mixing 400 μl type I collagen at 2 mg/ml with 150 μl RPMI 5×, 15 μl NaOH 1 N, 100 μl DMEM with 10% fetal calf serum and BZR cell suspension at 13 × 10^4 ^cells/ml. 150 μl of this mixture was added over the first layer and 1.5 ml of DMEM was placed into the basal compartment of the chamber that was thereafter maintained for 24 hours at 37°C. To test the effect of EGCG, the serum free DMEM medium in the baso-lateral compartment was complemented with EGCG at 5 μg/ml or 7.5 μg/ml

### 3D time-lapse videomicroscopy

Using the same microscope as for the 2D migration assay, image sequences of the cells within the collagen gel were recorded every hour at 110 depth levels (3 μm between each depth level) at 20× magnification. To quantify cell migration, we performed interactive tracking of cell positions in a four-dimensional dataset, as previously described [26]. Once the coordinates (x_ij_, y_ij_, z_ij_, t_j_) of every cell *i *at each *j *time setting are recorded in a data file, all the trajectories are known and parameters can be deduced. We measured the cell trajectory length in the horizontal plane (xy), in the vertical direction (z) and the total length of the trajectory (l). It was also useful to visualize these trajectories in the corresponding 3D space (X, Y, Z).

### RT-PCR Analysis

Total RNA extraction from subconfluent BZR cells was performed with the High Pure RNA isolation kit (Roche Diagnostics, Meylan, France). RT-PCR was performed with 4 ng/μl of total RNA using the GeneAmp Thermostable RNA PCR kit (Perkin-Elmer, Foster City, CA) and pairs of primers for human MMP-2, MMP-9, MT1-MMP, u-PA and for 28S rRNA (Eurogentec, Seraing, Belgium). Forward and reverse primers for human MMP-2, MMP9, MT1-MMP, u-PA and 28S were designed as follows:

MMP-2 primers (forward 5'-GGCTGGTCAGTGGCTTGGGGTA-3', reverse5'-AGATCTTCTTCTTC AAGGACCGGTT-3'),

MMP9 primers (forward 5'-GCGGAGATTGGGAACCAGCTGTA-3', reverse 5'-GACGCGCCTGTGTACACCCAACA-3'),

MT1-MMP primers (forward 5'-GGATACCCAATGCCCATTGGCCA-3', reverse 5'-CCATTGGGCATCCAGAAGAGAGC-3'),

u-PA primers (forward 5'-CTGTAATACGACTCACTATAGGGGGCACCGG-3', reverse 5'-TCCGGATAGAGATAGTCGGTGTGGTGAGCAAG-3'),

28S primers (forward 5'-GTTCACCCACTAATAGGGAACGTGA-3', reverse 5'-GGATTCTGACTTAGAGGCGTTCAGT-3').

Reverse transcription was performed at 70°C for 15 minutes. Amplification cycles were as follows: 15 seconds at 94°C, 20 seconds at 68°C, and 10 seconds at 72°C. Twenty one cycles were allowed for MMP-2 amplification, 30 cycles for MMP9 amplification, 20 cycles for MT1-MMP amplification, 26 cycles for u-PA amplification, 12 cycles for 28S amplification. Products were separated on acrylamide gels, stained with SYBR Gold (Invitrogen, Cergy Pontoise, France), and images were recorded by fluorimetric scanning (LAS-1000, Fuji, Stamford, CT).

### Zymography analysis

The BZR cell line was cultured in 12-well plates (10^4 ^cells per well). After 48 h of incubation, the medium was changed to serum-free medium and EGCG was added at different concentrations: 0, 5 or 7.5 μg/ml. After 18 h of incubation, the medium conditioned by the BZR was centrifuged. Samples were separated on a 10% polyacrylamide SDS gel containing 0.1% (w/v) gelatine (Sigma Aldrich, Saint-Quentin Fallavier, France). Electrophoresis was carried out at the constant current of 40 mA. The gel was washed for 1 hour at room temperature in a 2% (v/v) Triton X-100 solution, transferred to a 50 mmol/L Tris-HCl/10 mmol/L CaCl_2 _(pH 7.6) buffer and incubated overnight at 37°C. The gel was stained for 30 minutes in a 0.1% (w/v) Coomassie blue (G250)/45% (v/v) methanol/10% (v/v) acetic acid solution and de-stained in 10% (v/v) acetic acid/20% (v/v) methanol. Proteolytic activity was semi-quantified by densitometric scanning of the bands (LAS-1000, Fuji).

### Effect of EGCG on vimentin

We used the human breast cell line MCF10A in an *in vitro *model of cell migration. This model consisted in plating 5 × 10^4 ^cells inside a 6-mm glass ring placed in the middle of a collagen-coated coverslip [30]. Twenty four hours after plating, the glass ring was removed and the cells were covered with growth medium. The cells at the periphery of the culture were left to migrate for 24 h, then they were incubated with EGCG at 0, 5, or 7.5 μg/ml for another 24 h period. The migratory speeds were measured for 1 h as previously described and the cells were fixed in cold methanol for 10 min at -20°C. The coverslips were then saturated for 30 min with 3% bovine serum albumin in PBS. After intermediate washes in PBS, monolayers were successively incubated for 1 h with a monoclonal antibody to vimentin (clone Vim 3B4; Dako, Glostrup, Denmark), with biotinylated sheep anti-mouse antibody and with Alexa Fluor^® ^488-conjugated streptavidin (Dako). Coverslips were mounted with aqua polymount antifading solution (Polysciences, Warrington, PA) onto glass slides and observed under a fluorescence microscope at x10 or x63 magnification (AxioImager, Zeiss, Le Pecq, France).

### Data analysis

Values were reported as mean ± SD from at least 3 different experiments. Student's t-test was used for comparisons between groups and differences were considered to be statistically significant with *P *values less than 0.05.

## Results

### Effect of EGCG on cell death

**Figure 2:**
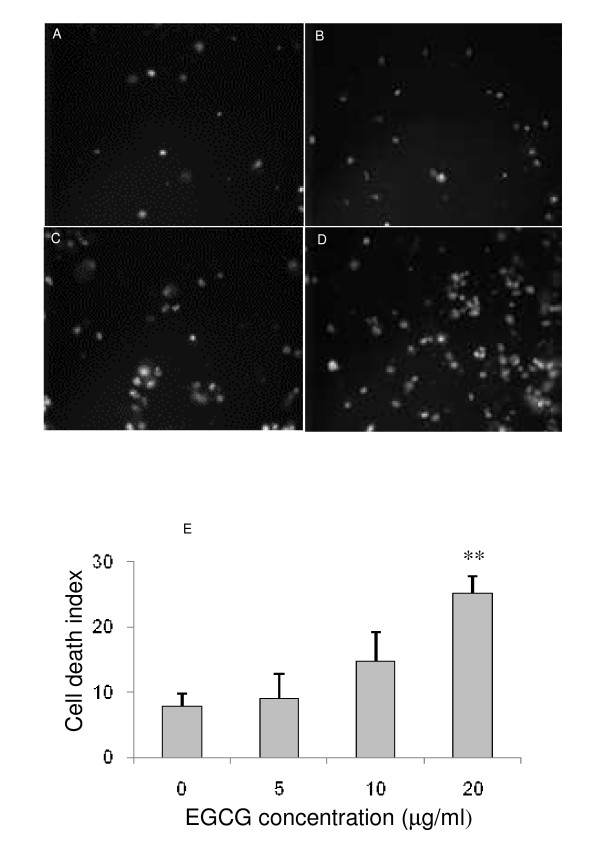
**Effect of EGCG on cell death**. Fluorescent images representing the effect of increasing concentrations of EGCG on cell death. The fluorescent probe propidium iodide was used to visualize dead cells. Compared to control (A) or to 5 μg/ml (B) and 10 μg/ml (C) of EGCG, we observed that 20 μg/ml (D) of EGCG induced a significant (p < 0.01) increase in the dead cell number (E).

To visualize the effect of EGCG on cell death, we used the fluorescent probe propidium iodide that specifically tags the nucleus of necrotic cells. Typical images are shown in figure 2. Cells were incubated without EGCG (figure 2A), or with EGCG at 5 μg/ml (figure 2B), 10 μg/ml (figure 2C), or 20 μg/ml (figure 2D). We observed a dose-dependent increase in the number of positive cell nuclei and this increase became significant (p < 0.01) in presence of 20 μg/ml of EGCG (figure 2E).

### 2D analysis of BZR trajectories in relation with cell migration speed

**Figure 3:**
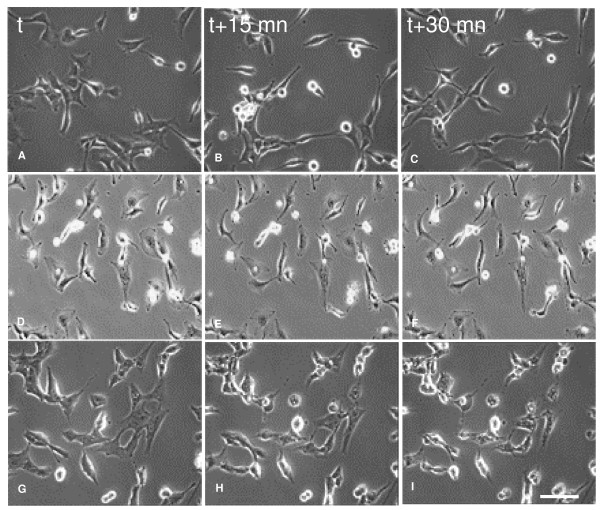
**Phase contrast images of BZR cells**. Phase contrast images of BZR cells in control medium or in medium with 5 or 7.5 μg/ml of EGCG. The images were recorded every 15 min. In absence of EGCG, evident alterations of the cell morphology were observed in parallel with the cell displacement (A, B, C). Cell shape modifications and cell movements were less important in presence of 5 μg/ml of EGCG (D, E, F) and were almost completely inhibited in presence of 7.5 μg/ml of EGCG. Scale bar = 50 μm.

**Figure 4:**
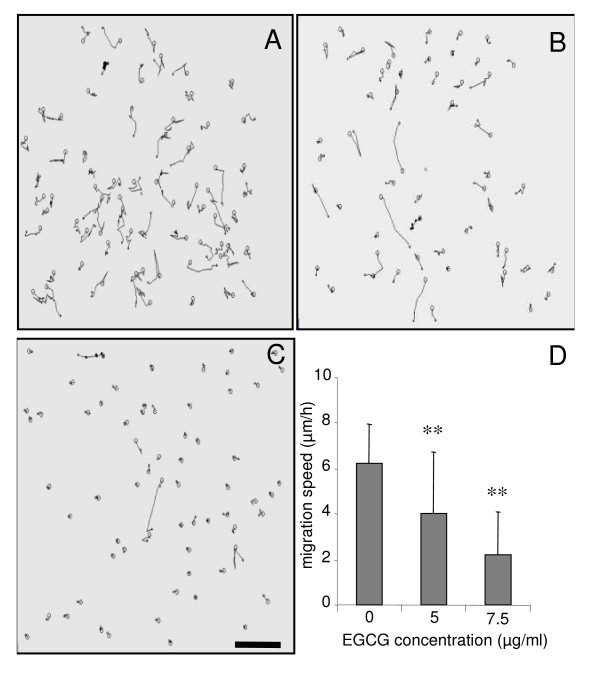
**Two-dimensional representation of the cell trajectories**. Trajectories of control BZR cells (A), BZR cells incubated with 5 μg/ml (B) or with 7.5 μg/ml (C) of EGCG for a 18 h migratory period. A significant (p < 0.01) decrease of the migration speed was observed when BZR cells were incubated with increasing concentrations of EGCG, compared with control BZR cells (D). Scale bar = 10 μm.

Observation of time lapse movies built from the phase contrast images recorded every 15 minutes for 18 hours showed an increasing inhibition of BZR migration in parallel with the increase of EGCG concentration. Time-lapse images recorded every 15 min showed that BZR cells in absence of EGCG continuously modified their shape and acquired an elongated morphology corresponding to a migratory phenotype (figure 3A, B, C). At the opposite, the incubation of BZR cells with EGCG at 5 μg/ml (figure 3D, E, F) or 7.5 μg/ml (figure 3G, H, I) induced the inhibition of cell shape modifications. From these time-lapse sequences, we quantified the cell migration speed and the results in figure 4 display the cell trajectories computed after 18 hours for the control, (figure 4A), with 5 μg/ml EGCG (figure 4B) and with 7.5 μg/ml EGCG (figure 4C). Quantification of the migration speed showed a significant (p < 0.01) and progressive decrease in presence of EGCG at 5 μg/ml and 7.5 μg/ml. This decrease reached 40% with 5 μg/ml and 68% with 7.5 μg/ml of EGCG as compared with the control (figure 4D).

### 3D analysis of BZR trajectories

**Figure 5:**
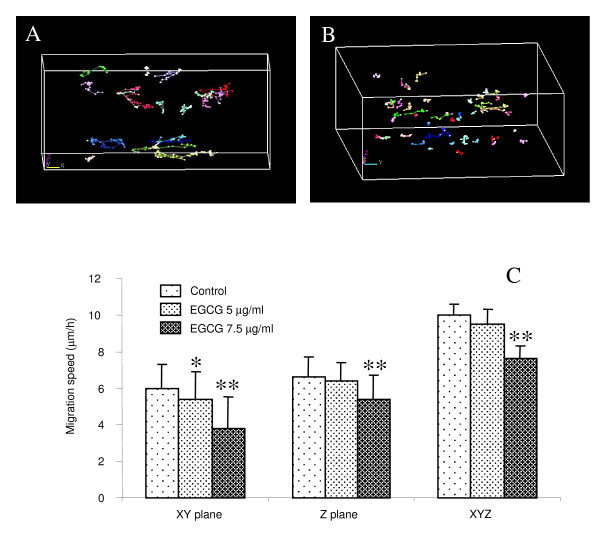
**Three-dimensional representation of the cell trajectories**. Trajectories of control BZR cells (A) and BZR cells incubated with 7.5 μg/ml of EGCG (B) for 18 h. Each color on the figure corresponds to different cells. A longer distance of migration was observed for control BZR cells compared with BZR cells treated with EGCG. A significantly lower (p < 0.05) migration speed along the xy direction was observed for BZR cells in presence of EGCG at 5 μg/ml. The presence of EGCG at 7.5 μg/ml in the lower compartment of the cell culture chamber significantly decreased (p < 0.01) BZR cell migration speed along the xy, z and xyz directions, compared with BZR cell migration speed in absence of EGCG (C).

Videomicroscopy and computational techniques were used to analyze the migratory behavior of cells and the effect of EGCG on 3D cell migration. Figure 5A displays the 3D trajectories of control BZR cells over 18 h of observation. The trajectories obtained under the same conditions with the BZR cells incubated with 7.5 μg/ml of EGCG are presented in figure 5B. We observed a higher trajectory length for control BZR cells compared to BZR cells incubated with EGCG. The migration parameters computed from these trajectories are summarized in Figure 5C: a significant decrease (p < 0.05) of the migration speed along the XY horizontal plane was observed for BZR cells in presence of EGCG at 5 μg/ml compared with control BZR cells. When incubated with EGCG at 7.5 μg/ml, a significantly (p < 0.01) higher decrease in the migration speed of BZR cells was observed along the XY horizontal plane, the Z plane and in the XYZ volume, compared with control BZR cells.

### RT-PCR and zymography analysis

**Figure 6:**
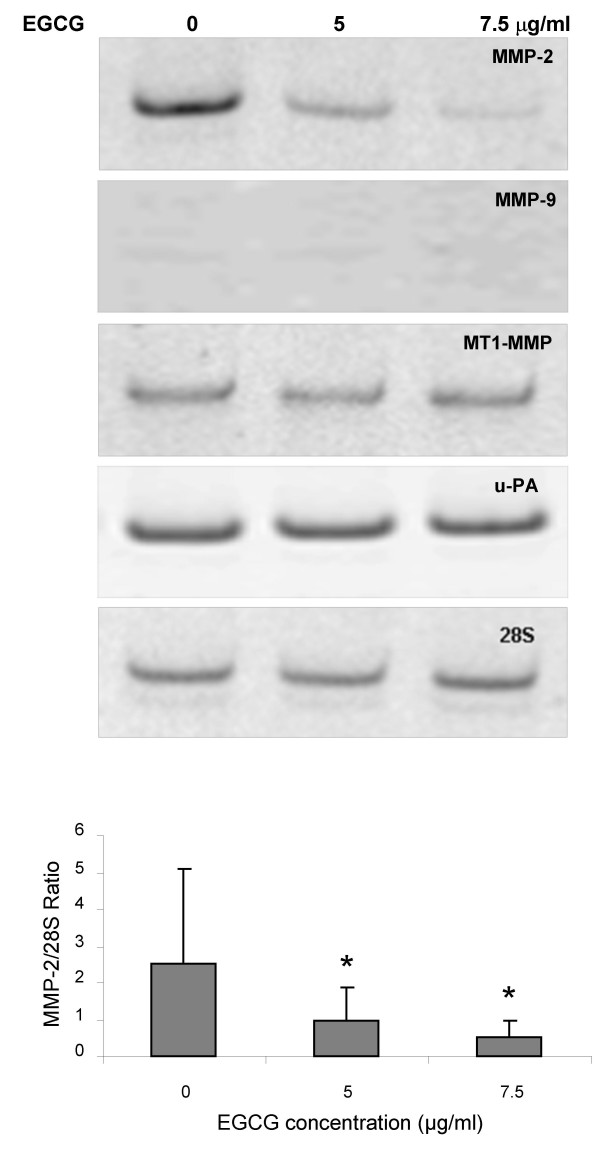
**mRNA expression**. mRNA expression for MMP2, MMP9, MT1-MMP and u-PA by BZR cells incubated with increasing concentrations of EGCG. A progressive inhibition of the mRNA level for MMP2 and no changes in MT1-MMP and u-PA mRNA level were observed in parallel with the increase of EGCG concentration. MMP9 expression was not detectable.

To evaluate the effect of EGCG on protease gene expression, we analyzed the mRNA amount of MMP-2, MMP-9, MT1-MMP and u-PA using semi-quantitative RT-PCR (figure 6). We observed a significant (p < 0.05) decrease of the MMP-2 transcript expression in a dose-dependent manner after 18 h of treatment with EGCG but we did not observe any significant change of the MT1-MMP and u-PA transcript expression. The level of MMP9 transcript expression was not detectable.

**Figure 7:**
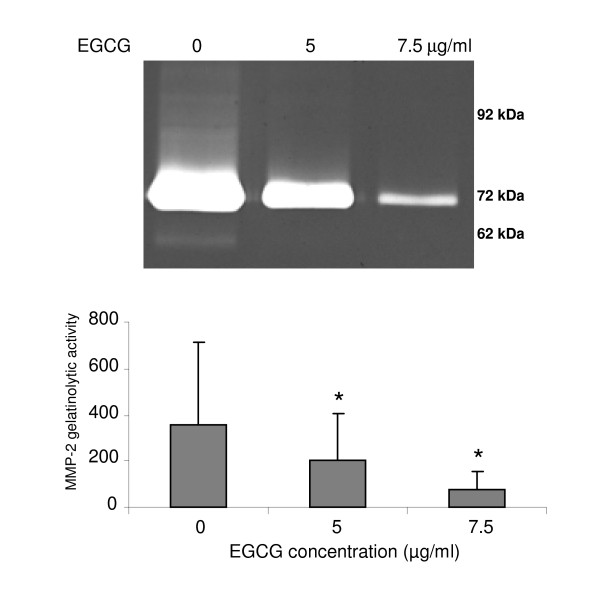
**zymography of the gelatinolytic activities of MMPs**. Analysis of the gelatinolytic activities of MMP9 (92 kDa), pro-MMP2 form (72 kDa) and MMP2 active form (62 kDa) of BZR cells incubated with different concentrations of EGCG (0, 5 and 7.5 μg/ml). A significant EGCG dose-dependent decrease (p < 0.05) of pro-MMP2 form was observed compared to control. Neither active MMP2 form, nor gelatinolytic activity for MMP9, were observed in presence of EGCG.

Zymography analysis shows a significant dose-dependent decrease (p < 0.05) of the active and latent form of MMP-2 after 18 hours of incubation with EGCG in comparison with the control (Figure 7). No enzymatic activity corresponding to MMP-9 was observed.

### Effect of EGCG on vimentin expression

**Figure 8:**
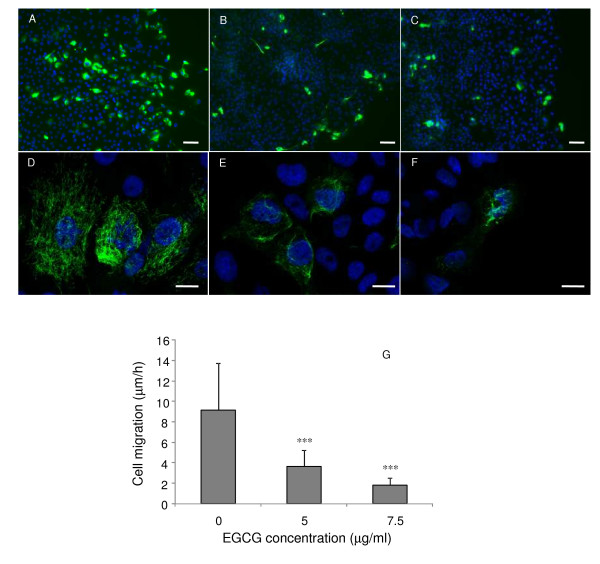
**Effect of EGCG on vimentin**. Immunolocalization of vimentin in control MCF10A cells (A, D) or MCF10A cells incubated with 5 (B, E) and 7.5 μg/ml (C, F) of EGCG. In presence of EGCG at 5 (B) or 7.5 μg/ml (C), we observed a decrease in the number of cells expressing vimentin. At higher magnification (D, E, F), alterations of the vimentin network were observed: less expression and more condensed (E, F) compared to control (D). Scale bar = 50 μm (A, B, C) or 20 μm (D, E F).

To examine the potential effect of EGCG on vimentin-dependent migration, we used the ring culture system that allowed the MCF10A cell line to specifically express vimentin in migratory cells at the periphery of the culture [30]. As shown in figure 8G, we observed that the incubation of migrating MCF10A cells with increasing concentrations of EGCG significantly decreased the cell migration speed. In parallel with the decrease in cell migration speed, we noticed different patterns of vimentin expression. In control experiments, most of the cells at the periphery of the ring culture system express vimentin (figure 8A). When the cells were incubated with increasing concentrations of EGCG, the number of cells expressing vimentin progressively decreased (figure 8B, C). Changes in vimentin organization induced by EGCG are shown in figure 8D, E, F. Untreated Cells were characterized by an homogeneous network of vimentin (figure 8D). Within 18 h of incubation with 5 μg/ml of EGCG, we observed alterations of the vimentin network that was less expressed and more condensed (figure 8E). In presence of 7.5 μg/ml of EGCG, cell shape changes were observed, in parallel with vimentin disorganization (figure 8F).

## Discussion

Previous studies have shown that EGCG had beneficial effects on cancer prevention and inhibition and that these effects were associated to a large number of mechanisms [10,16,31-33]. In most studies, the concentrations needed to observe these effects typically range from 0.5 to 50 μg/ml. EGCG represents approximately 200 mg in a brewed cup of tea, and in mice, for reaching a plasma concentration near to 5 μg/ml, the ingestion of 2000 mg/kg EGCG is necessary. EGCG delivered in the form of capsules (200 mg) has been reported to be effective in the patients with human papilloma virus-infected cervical lesions [34]. EGCG has been reported to inhibit cell migration or invasion in liver cancer cells [5], glioblastoma cells [3], vascular smooth muscle cells [35,36], pancreatic stellate cells [37] or during angiogenesis [38]. However, these latter studies were performed by using *in vitro *models similar to the well-known Boyden chamber assay by which the cell migration was evaluated by counting the number of cells present on the basal side of a porous membrane. To date, the effect of EGCG on the migration speed of tumor cells has not been investigated. We therefore used *in vitro *models of cell migration associated to computational techniques for studying the effect of EGCG on the migration of invasive cell lines. The effect of EGCG on cell migration was higher when cells were cultured in 2D systems (65% decrease in presence of 7.5 μg/ml of EGCG) compared with a 3D environment (25% decrease of the total distance in presence of 7.5 μg/ml of EGCG). This difference in EGCG effect on cell migration speed according to the culture model could be related to the cell-EGCG interaction that could be less effective in the collagen-rich environment used in the 3D culture model. To confirm that the EGCG acted exclusively on cell migration, in preliminary experiments, we also tested the apoptotic effect of EGCG on the BZR cell line and we did not observe significant cell death at EGCG concentrations lower than 10 μg/ml. This emphasizes the inhibitory effect of EGCG on cell migration.

In parallel with the decrease of the migration of cells incubated with EGCG, we observed alterations of the vimentin cytoskeleton network. Vimentin expression has been described in epithelial cells to be involved in pathological or physiological processes that require epithelial cell migration. In addition, data from Gilles et al [39] clearly demonstrated that vimentin expression was related to the migratory status of cells, suggesting that vimentin may play a fundamental role in cell migration. Moreover, vimentin expression was only found in human epithelial tumor cells lines displaying high invasive abilities. The BZR cells used in the present study constitutively expresses vimentin independently from their migratory status. To provide a direct link between the inhibitory effect of EGCG on vimentin and migration, we used the MCF10A cell line that has been reported to specifically express vimentin during migration [30]. We observed that the decreased cell migration induced by EGCG was accompanied by a decrease in vimentin expression and organization. We hypothesize that the alterations of the vimentin network induced by EGCG likely led to the decrease of cell migration. Our results are emphasized by those previously reported by Ermakova et al [40] who demonstrated that vimentin is a target for EGCG by inhibiting phosphorylation of vimentin. Although for most motile cells, cell movement is clearly dependent upon the dynamics of an actin microfilament system, intermediate filaments such as vimentin are also important in cell movement because they act to stiffen the internal cytoskeleton and thereby organize actin networks from which filopodia or lamellipodia polymerize outward [41].

Beside the effect of EGCG on vimentin organization, we observed an important inhibition of the expression and the gelatinolytic activity of matrix metalloproteases such as MMP-2 during the incubation with EGCG, but no change was observed concerning MT1-MMP expression. MMP-2 has been shown to be involved in tumor invasion *in vitro*. Indeed, MMP-2 overexpression has been associated not only with the invasive potential of many tumor cell lines *in vitro *[11,27,42] but also with the malignant phenotype *in vivo *[43,44]. Furthermore, many reports have indicated that increased MMP-2 activity was observed in human tumor cell lines displaying an invasive phenotype and was associated with the metastatic potential of breast and colon carcinomas, supporting the essential role of MMP-2 in tumor invasion. We demonstrated, in this study, that EGCG treatment inhibits the activation of MMP-2 associated with a decreased of migratory and invasive capacities of human bronchial tumor cells. We did not observed EGCG-induced variations in MT1-MMP mRNA level. These results are similar to those reported by El Bedoui et al [45] who demonstrated inhibition of MT1-MMP activity by green tea extracts rather than changes in MT1-MMP mRNA and protein expression. Accordingly, it has been previously demonstrated that the activity of MT1-MMP and of the active form of MMP-2 in the medium of human endothelial cells was decreased in presence of EGCG [46] and that the consequence of the inhibitory activity of metalloproteases was a blocking of tumor cell invasion [2]. EGCG has been shown to affect MMPs both directly and indirectly. Recently, EGCG has been reported to inhibit activating protein-1 (AP-1) that regulates MMP expression. In another way, EGCG could also inhibit the proMMP-2 protein secretion by perturbing the general intracellular vesicular trafficking [16]. A contradictory result is observed for MMP9 which is not expressed under the present experimental conditions, but has been reported in previous experiments [47] to be expressed by BZR cells. This apparent discrepancy in the results could be related to differences in culture conditions. In the present work we used cultures at 50 to 60% of confluency (which is a necessary condition for accurate measurement of cell migration), whereas the cultures were subconfluent in the previous experiments. In the same manner, we did not detect any variation in u-PA. These results are apparently contradictory with those reported by Jankun et al [48] who noticed an inhibitory effect on u-PA at EGCG concentrations ranging between 1 mM to 10 mM, which are much higher than the concentrations used in the present study (5 to 15 μM).

## Conclusion

Taken together our results demonstrate that beside their well-known antiproliferative effects, green tea catechins are also able to inhibit the migration of bronchial tumor cells and could therefore be attractive candidates to treat tumor invasion.

## Competing interests

The authors declare that they have no competing interest.

## Authors' contributions

SH carried out the BZR cell cultures, videomicroscopic recordings, quantification and drafted the manuscript. AB performed MCF10 cell cultures and migration experiments. BNR participated in RT-PCR, zymography and helped to draft the manuscript. MM participated in RT-PCR and performed immunofluorescence. CT participated in the development of images analysis techniques. JC developed images analysis techniques. MP participated in RT-PCR, zymography and helped to draft the manuscript. PB and JMZ conceived the study, participated in its design, coordination and helped to draft the manuscript. All authors read and approved the final manuscript.
